# Circulating magnesium levels and incidence of coronary heart diseases, hypertension, and type 2 diabetes mellitus: a meta-analysis of prospective cohort studies

**DOI:** 10.1186/s12937-017-0280-3

**Published:** 2017-09-19

**Authors:** Jiang Wu, Pengcheng Xun, Qingya Tang, Wei Cai, Ka He

**Affiliations:** 1 Department of Clinical Nutrition, Xin Hua Hospital, School of Medicine, Shanghai Jiao Tong University, No. 1665, Kongjiang Rd, Shanghai, China; 2 Shanghai Key Laboratory of Pediatric Gastroenterology and Nutrition, Shanghai, China; 3 Shanghai Institute for Pediatric Research, Shanghai, China; 4 Department of Epidemiology and Biostatistics, School of Public Health-Bloomington, Indiana University, 1025 E. Seventh Street, C042, Bloomington, Indiana 47405 USA; 5 Department of Pediatric Surgery, Xin Hua Hospital, School of Medicine, Shanghai Jiao Tong University, Shanghai, China

**Keywords:** Magnesium, Coronary heart disease, Hypertension, Type 2 diabetes, Meta-analysis

## Abstract

**Background:**

Data on the associations between circulating magnesium (Mg) levels and incidence of coronary heart diseases (CHD), hypertension, and type 2 diabetes mellitus (T2DM) are inconsistent and inconclusive. The aim of this study was to examine circulating Mg levels in relation to incidence of CHD, hypertension, and T2DM.

**Methods:**

Prospective cohort studies published before May 2017 were searched through PubMed, EmBase, SCOPUS, and Google Scholar. A total of 11 studies that reported multivariable-adjusted associations of interest were identified. Information on the characteristics of study and participants, exposure, main outcomes, risk estimates, and cofounders was extracted and analyzed.

**Results:**

Of the 11 included studies, 5 reported results on CHD (38,808 individuals [4437 cases] with an average 10.5-year follow-up), 3 on hypertension (14,876 participants [3149 cases] with a 6.7-year follow-up), and 4 on T2DM (31,284 participants [2680 cases] with an 8.8-year follow-up). Comparing the highest to the lowest category of circulating Mg concentration, the pooled relative risks [RRs] (95% confidence intervals [CIs]) were 0.86 (0.74, 0.996), 0.91 (0.80, 1.02), and 0.64 (0.50, 0.81) for incidence of CHD, hypertension, and T2DM, respectively. Every 0.1 mmol/L increment in circulating Mg levels was associated with 4% (RR, 0.96; 95% CI: 0.94, 0.99) reduction in hypertension incidence. No significant linear association was found between circulating Mg levels and incidence of CHD (RR, 0.89; 95% CI: 0.77, 1.03) and T2DM (RR, 0.90; 95% CI: 0.81, 1.002). The observed associations of interest were sensitive to exclusion of individual studies.

**Conclusions:**

Findings in this meta-analysis suggest that circulating Mg levels are inversely associated with incidence of CHD, hypertension, and T2DM. Additional studies are needed to provide more solid evidence and identify the optimal range of circulating Mg concentration with respect to primary prevention of CHD, hypertension, and T2DM.

**Electronic supplementary material:**

The online version of this article (10.1186/s12937-017-0280-3) contains supplementary material, which is available to authorized users.

## Introduction

Studies suggest that coronary heart diseases (CHD), hypertension, and type 2 diabetes mellitus (T2DM) are comorbidities and the major risk factors of mortality [1, 2]. Incidences of these chronic diseases are growing rapidly [3–5], and identifying modifiable risk factors is crucial to the prevention of these diseases.

Magnesium (Mg) is the second most predominant intracellular electrolyte, following after potassium. Mg serves as an important cofactor in many essential enzymatic reactions involved in glucose metabolism and several essential physiological processes, including modulating vascular smooth muscle tone and endothelial cell function [6–8]. The role of Mg in the development and progress of T2DM and cardiovascular diseases (CVD) has drawn researchers’ attention in the recent decades [9–13]. Several large prospective cohort studies have shown that low Mg intake is associated with incidence of T2DM and CVD [11, 12, 14–16]. Notably, because Mg intake was usually assessed based on self-administered food frequency questionnaires [11, 15, 16], misclassification is inevitable. In addition, dietary Mg could not represent the accurate amount of Mg intake without considering the substantial loss during food processing and cooking [17]. Moreover, the health impact of Mg is difficult to be distinguished from intake of other nutrients such as calcium, potassium, phosphorus, and fiber.

Serum or plasma Mg concentration is the most commonly used biomarker to assess Mg metabolism abnormality in clinical practice. It reflects not only the dietary intake, but also the intestinal absorption, renal reabsorption and excretion, and hormone regulation. Healthy individuals maintain quite stable circulating Mg levels, except for in cases of Mg deficiency. Of note, the preponderance of epidemiological and clinical information relevant to chronic disease associations with Mg status is derived from serum total Mg levels, which reasonably approximates ionized (free) Mg concentration [18]. Other biomarkers such as 24-h urine Mg, red blood cell Mg, and ionized Mg were often restricted in large-scale epidemiological studies due to budget and ethical considerations. A recently published meta-analysis on randomized controlled trials revealed a dose-response relationship of oral Mg supplementation with circulating Mg levels [19], which supports circulating Mg as a reasonable biomarker of Mg status.

Many studies suggest that low circulating Mg levels are associated with insulin resistance [12, 20, 21], which is a known risk factor of T2DM, CHD and hypertension [22]. A number of prospective cohort studies have examined the association between circulating Mg levels and incidence of CHD [23–27], hypertension [26, 28, 29], or T2DM [11, 30–32], but the findings are inconsistent and inconclusive. One recent meta-analysis examined both dietary intake of Mg and circulating Mg levels with the risk of CHD found an inverse association [33]. Earlier, two meta-analyses found that circulating Mg concentrations were inversely related to the risk of CVD [13, 34]. However, dietary intake of Mg is subject to measurement error. Also, CVD includes a number of outcomes that may have different pathophysiology, e.g., ischemic vs. hemorrhagic stroke. In addition, studies suggest that CHD, hypertension, and T2DM are comorbidities and the major risk factors of mortality. Of note, these three chronic diseases share the same risk factor - insulin resistance, which is closely related to circulating Mg levels. Therefore, we aimed to quantitatively summarize the literature by conducting a meta-analysis of prospective cohort studies on the associations of circulating Mg levels with incidence of CHD, hypertension, and T2DM.

## Methods

This meta-analysis was conducted following the preferred reporting items for systematic reviews and meta-analyses (PRISMA) guideline [35]. The completed PRISMA checklist is available in Additional file 1: Table S1 (see supplemental materials).

### Data sources and search strategy

We conducted a systematic literature review to identify all the studies on the associations between circulating Mg levels and incidence of CHD, hypertension, or T2DM through May 2017. We first searched the electronic database of PubMed (https://www.ncbi.nlm.nih.gov/pubmed) using the following MeSH terms: (“micronutrients” OR “magnesium” OR “magnesium deficiency”) AND (“cardiovascular disease” OR “myocardial infarction” OR “ischemic heart disease” OR “hypertension” OR “blood pressure” OR “coronary heart disease” OR “diabetes mellitus” OR “hyperglycemia” OR “insulin resistance”) AND (“cohort studies” or “follow-up studies” OR “longitudinal studies” OR “prospective studies”). We further reviewed EmBase (http://www.elsevier.com/online-tools/embase), SCOPUS (https://www.scopus.com/) and Google Scholar (http://scholar.google.com/). In addition, we identified relevant articles by manually searching the references of the retrieved studies and review articles.

### Study selection

Articles were selected if they met the following criteria: published in English; had a prospective design; evaluated the association between baseline circulating Mg levels and CHD, hypertension, or T2DM; and reported estimated relative risks (RRs), hazard ratios (HRs), or odds ratios (ORs) with 95% confidence intervals (CIs) or these data could be calculated from the available information. In the included studies, CHD was defined as any coronary heart disease, including ischemic heart disease (IHD) incidence or death, angina, myocardial infarction and sudden cardiac death. Hypertension was defined as a systolic blood pressure ≥ 140 mmHg, or a diastolic blood pressure ≥ 90 mmHg, or the use of anti-hypertensive drugs. T2DM was defined based on the blood glucose levels (fasting plasma glucose ≥7.0 mmol/l, or, non-fasting or 2 h post-load glucose ≥11.1 mmol/l) or the use of anti-diabetic drugs. New-onset CHD, hypertension and T2DM was determined by medical records, self-reports or death certificates. All the articles were identified by an initial screen of abstracts, followed by a full-text review, which was independently conducted by two investigators (JW and PX).

### Data extraction and quality assessment

The relevant data were independently extracted by two investigators (JW and PX) using a standardized form, and disagreements were resolved by consensus after discussion with the third investigator (KH). The following information was extracted: study characteristics (study name, publication year, authors, country where the study was conducted, study design, sample size, and follow-up time), participants’ characteristics (age at baseline, race, proportion of male gender, major covariates), exposure (method of assessment, classification), main outcomes, and estimated RRs with 95% CIs for corresponding categories and/or continuous exposure. If more than one multivariate model was reported, the estimates with full adjustment for potential confounders were extracted.

The quality of included studies was ascertained with the Newcastle-Ottawa quality assessment scale (NOS) [36] by 2 investigators (JW and PX) independently. Any disagreement was solved by group discussion with the third investigator (KH). This assessment allowed a total score of up to 9 points. The NOS for cohort studies was divided into three groups: selection of cohort (4 points), comparability of cohort (2 points), and assessment of outcome (3 points). The quality of study was considered high or moderate if the sum score was ≥8 points or between 5 and 7 points, respectively.

### Statistical methods

All analyses were performed by using STATA statistical software (Version 13.0; STATA Corporation LP, College Station, Texas, US). Unless otherwise specified, a value of *P* ≤ 0.05 was considered statistically significant.

In this meta-analysis, we used RRs and 95% CIs as a measure of the effect size for all studies. Hazards ratio was considered RR directly, and OR was considered RR in the main analysis and was transformed into RR in a sensitivity analysis by using the formula: RR = OR/[(1-*P*_0_) + OR*P_0_)], where *P*_0_ indicated incidence of the outcome of interest in the reference group [37]. RRs and 95% CIs transformed to their natural logarithms (ln) were used to compute the corresponding standard errors. RRs and 95% CIs were converted to per 0.1 mmol/L increment consistently to describe the linear associations of interest regardless of the original unit (mEq/L, mmol/L or mg/dL) for Mg levels. If a study did not provide the linear association of circulating Mg concentration with the outcomes of interest, we estimated it based on the categorical associations by using Greenland and Longnecker’s method, if the person-time of participants as well as cases were reported for each subgroup of Mg levels [38], or using variance-weighted least squares linear regression if they were not reported. If the highest or the lowest group of Mg levels was an open range, then its upper or lower limit was estimated by assuming its range as the same width as the adjacent category.

We pooled RR estimates separately for each outcome using a random-effects model. We evaluated the statistical heterogeneity of the RRs using Cochran’s *Q* test with a significance level of 0.10, and quantified the heterogeneity using the *I* ^2^ statistic with a value of 0–25%, 26–50%, 51–75%, or >75% denoting very low, low, moderate, and high degrees of heterogeneity, respectively. Publication bias was visually examined by funnel plots, and statistically assessed by using Egger’s regression asymmetry test and Begg’s rank correlation test, with a significance level of 0.10. If publication bias existed, the Duval and Tweedie nonparametric “trim and fill” method was used to adjust for the pooled results [39].

The average of follow-up year was calculated as the sum of person-year divided by total number of participants. Sensitivity analyses were conducted to evaluate the influence of replacing the random-effects model with the fixed-effects model and the influence of a single study on the overall association by calculating the pooled estimates while omitting one primary study at each time. In addition, OR was transformed to RR in a sensitivity analysis.

## Results

### Literature search

**Fig. 1:**
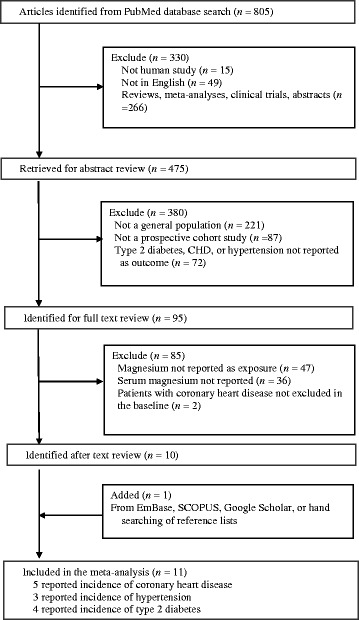
Study selection process. Articles were identified by searches of PubMed (http://www.ncbi.nlm.nih.gov/pubmed), EmBase (http://www.elsevier.com/online-tools/embase), SCOPUS (https://www.scopus.com/) and Google Scholar (http://scholar.google.com)

As shown in Fig. 1, we retrieved 805 relevant articles from PubMed. Of them, 795 articles were excluded for one of the following reasons: 1) not a human study; 2) a review/meta-analysis, editorial, or abstract; 3) not published in English; 4) not conducted in the general population, but in patients (e.g., diabetic patients); 5) not relating circulating Mg levels to an outcome of interest; 6) not a prospective cohort design; or 7) did not exclude prevalent cases at baseline. In addition, we found 1 additional articles by searching Google Scholar. Thus, 11 studies were identified and included in this meta-analysis.

### Characteristics of included studies

Table 1 summarizes the characteristics of the included studies, all of which had a prospective cohort design and had participants without a prior diagnosed outcome of interest at baseline. Some studies estimated RRs/HRs/ORs with 95% CIs for men and women separately [24, 28], or for blacks and whites independently [11], and these were counted as separate cohorts in the meta-analysis. Of these 11 prospective studies, 5 studies (6 cohorts) reported results on CHD with 45,808 individuals and 4437 cases during an average of 10.5 years of follow-up [23–27], 3 studies (4 cohorts) on hypertension with 14,876 participants and 3149 cases during an average of 6.7 years of follow-up [26, 28, 29], and 4 studies (5 cohorts) on T2DM with 31,284 participants and 2680 cases during an average of 8.8 years of follow-up [11, 30–32].

**Table 1:** Characteristics of the 11 prospective studies included in the meta-analysis

Source	Age at baseline (year)	Men (%)	Duration of follow-up (year)	No. of participants /events	Exposure categories	Exposure assessment	Outcome and its assessment	Adjusted variables	Main results	Study quality
Coronary heart diseases										
Gartside et al. (1995) [23], The NHANES Study, USA	25–74	NA	10	8251/492	Baseline serum magnesium level (mEq/L): <1.62; 1.62- < 1.74; ≥1.74.	NA	CHD events were determined by hospital records and death certificates (ICD-9 codes 410–414 for CHD).	Age, gender, quetelet index, exercise, sedimentation rate, dietary iron, maximum weight, cigarette smoking, exercise, riboflavin, and alcohol consumption at baseline.	RR (95% CI):1.00 (Referent);0.96 (0.78, 1.19);0.68 (0.54, 0.87).	9
Liao et al. _female. (1998) [24], The ARIC Study, USA	45–64	0	5.2	7767/96	Baseline serum magnesium level (mEq/L): ≤1.5; 1.6; 1.7; 1.8.	Serum magnesium was measured using the metallo-chromic dye calmagite, based on the procedure of Gindler and Heth.	CHD incidence was ascertained by self-report, medical records, and death certificates. Out-of-hospital deaths were confirmed both by the death certificate and an interview with the next of kin and questionnaires completed by the patient’s physicians.	Age, race, field center, waist/hip ratio, smoking status, ethanol drinking, education, sports index, fibrinogen, total and HDL cholesterol, triglycerides, diuretic use, hormone replacement, SBP, and diabetes status.	RR (95% CI):1.00 (Referent);1.02 (0.61, 1.71);0.59 (0.30, 1.20);0.55 (0.27, 1.14).*P*trend = 0.047.	9
Liao et al. _male. (1998) [24], The ARIC Study, USA	45–64	100	5.2	6155/223	Baseline serum magnesium level (mEq/L): ≤1.5; 1.6; 1.7; 1.8.	Serum magnesium was measured using the metallo-chromic dye calmagite, based on the procedure of Gindler and Heth.	CHD incidence was ascertained by self-report, medical records, and death certificates. Out-of-hospital deaths were confirmed both by the death certificate and an interview with the next of kin and questionnaires completed by the patient’s physicians.	Age, race, field center, waist/hip ratio, smoking status, ethanol drinking, education, sports index, fibrinogen, total and HDL cholesterol, triglycerides, diuretic use, SBP, and diabetes status.	RR (95% CI):1.00 (Referent);1.48 (1.03, 2.13);1.08 (0.72, 1.61);0.84 (0.53, 1.31).*P*trend = 0.23.	9
Ford et al. (1999) [25], The NHANES Study, USA	25–74	40.1	15.5	12,340/2637	Baseline serum magnesium level (mmol/L): 0.41- < 0.80; 0.80- < 0.84; 0.84- < 0.89; 0.89–1.45.	Atomic absorption spectrophotometry.	IHD events were determined by hospital records (ICD-9-CM codes 410–414).-	Age, gender, race, education, smoking, cholesterol, SBP, antihypertensive medication, self-reported diabetes, BMI, leisure-time and non-leisure time physical activity, and alcohol consumption.	HR (95% CI):1.00 (Referent);0.95 (0.79, 1.14);0.87 (0.73, 1.04);0.92 (0.79, 1.07).	9
Khan et al. (2010) [26], The Framingham offspring Study, USA	44.3 ± 10.0	48.3	20	3531/554	Baseline serum magnesium level (mg/dL): 1.40–1.77; 1.77–1.88; 1.88–1.98; 1.98–2.50.	Serum magnesium was measured using standard colorimetric assay.	CVD event was defined as angina pectoris, coronary insufficiency (prolonged angina with documented ECG changes), MI, stroke or transient ischemic attack, heart failure, intermittent claudication, or death secondary to CVD.	Age, sex, BMI, diabetes, SBP, total/HDL cholesterol ratio, smoking, hemoglobin, albumin, and estimated GFR.	HR (95% CI):1.00 (Referent);1.09 (0.86, 1.37);0.88 (0.69, 1.13);0.91 (0.72, 1.17).*P*trend = 0.23.Continuous(↑ 0.15 mg/dL):0.83 (0.49, 1.40).	9
Joosten et al. (2013 a) [27], The PREVEND study, The Netherlands	28–75	49	8.1	7764/435	Baseline plasma magnesium level (mmol/L): <0.77; 0.77–0.79; 0.80–0.82; 0.83–0.85; >0.85.	Plasma magnesium was measured by a xylidyl blue method.	Incident IHD was defined as follows: acute myocardial infarction (ICD-10 code I21), hospitalization for other acute ischemic heart disease (ICD-10 code I24), coronary artery bypassing grafting, or percutaneous transluminal coronary angioplasty.	Age, sex, BMI, smoking, diabetes, ratio of total to HDL cholesterol, parenteral history of IHD, alcohol intake, plasma levels of sodium, potassium, and calcium.	HR (95% CI):1.06 (0.79, 1.43);0.90 (0.66, 1.22);1.00 (Referent);1.08 (0.80, 1.45);1.07 (0.80, 1.43).*P*trend = 0.59.	9
Hypertension										
Peacock et al. _female. (1999) [28], The ARIC Study, USA	52.8 (45–64)	0	6	4190/822	Baseline serum magnesium levels in quartiles (mEq/L): 0.7–1.5; 1.6; 1.7; 1.8–2.3.	Serum magnesium was measured using the metallo-chromic dye calmagite, based on the procedure of Gindler and Heth.	Incident hypertension was defined as the new occurrence of SBP ≥ 140 mmHg or DBP ≥ 90 mmHg, or currently taking antihypertensive medication at the follow up visits.	Age, race, field center, BMI, waist/hip ratio, diabetes, education, family history of hypertension, leisure activity score, hormone replacement therapy, and baseline SBP.	OR (95% CI):1.00 (Referent);0.82 (0.64, 1.05);0.93 (0.73, 1.19);0.76 (0.58, 0.99).*P*trend = 0.11.	9
Peacock et al. _male. (1999) [28], The ARIC Study, USA	53.5 (45–64)	100	6	3541/755	Baseline serum magnesium levels in quartiles (mEq/L): 0.7–1.5; 1.6; 1.7; 1.8–2.3.	Serum magnesium was measured using the metallo-chromic dye calmagite, based on the procedure of Gindler and Heth.	Incident hypertension was defined as the new occurrence of SBP ≥ 140 mmHg or DBP ≥ 90 mmHg, or currently taking antihypertensive medication at the follow up visits.	Age, race, field center, BMI, waist/hip ratio, diabetes, education, family history of hypertension, leisure activity score, and baseline SBP.	OR (95% CI):1.00 (Referent);0.91 (0.70, 1.19);0.92 (0.71, 1.19);0.90 (0.68, 1.18).*P*trend = 0.50.	9
Khan et al. (2010) [26], The Framingham offspring Study, USA	42.5 ± 9.5	45.0	8	2520/551	Baseline serum magnesium level (mg/dL): 1.40–1.77; 1.77–1.88; 1.88–1.98; 1.98–2.50.	Serum magnesium was measured using standard colorimetric assay.	Incident hypertension was defined as the new occurrence of SBP ≥ 140 mmHg or DBP ≥ 90 mmHg or currently taking antihypertensive medication, including diuretics.	Age, sex, BMI, diabetes, systolic blood pressure, total/HDL cholesterol ratio, smoking, hemoglobin, albumin, and eGFR.	HR (95% CI):1.00 (Referent);0.97 (0.72, 1.31);0.96 (0.70, 1.32);1.03 (0.75, 1.41).*P*trend = 0.89.Continuous(↑ 0.15 mg/dL):1.03 (0.92, 1.15).	9
Joosten et al. (2013 b) [29], The PREVEND study, The Netherlands	28–75	45.3	7.6	4625/1021	Baseline plasma magnesium level (mmol/L): 0.55–0.77; 0.78–0.80; 0.81–0.84; 0.85–1.04.	Plasma magnesium was measured by a xylidyl blue method.	Incident hypertension was defined as the new occurrence of SBP ≥ 140 mmHg, DBP ≥ ≥ 90 mmHg, or initiation of antihypertensive medication.	Age, sex, BMI, smoking, diabetes, parenteral history of hypertension, alcohol intake, study design, and plasma levels of sodium, potassium, and calcium.	HR (95% CI):1.00 (Referent);1.00 (0.84, 1.20);0.92 (0.77, 1.09);0.94 (0.79, 1.12).Continuous(mmol/L):0.94 (0.83, 1.05).	9
Diabetes mellitus										
Kao et al. _black. (1999) [11], The ARIC Study, USA	53.0	36.3	5.3	2622/367	Baseline serum magnesium level (mEq/L): 0.50–1.40; 1.50; 1.60; 1.70; 1.80; 1.90–2.60.	Serum magnesium was measured using the metallo-chromic dyecalmagite, based on the procedure of Gindler and Heth.	Diabetes mellitus was defined as the presence of any of the following: 1) fasting glucose levels ≥7.0 mmol/L; 2) non-fasting glucose levels ≥11.1 mmol/L; 3) current use of diabetic medication; 4) a positive response to the question “Has a doctor ever told you that you had diabetes?” Individuals without diabetes at baseline who met any of these conditions at follow-up were considered to have incident cases of diabetes.	Age, gender, education, BMI, family history of diabetes, waist/hip ratio, sports index, alcohol consumption, diuretic use, serum calcium and potassium levels, and fasting insulin and glucose levels at baseline.	OR (95% CI):0.90 (0.48, 1.68);0.82 (0.45, 1.51);1.01 (0.56, 1.83);0.94 (0.51, 1.73);0.94 (0.48, 1.81);1.00 (Referent).*P*trend = 0.71.	9
Kao et al. _white. (1999) [11], The ARIC Study, USA	54.2	46.0	5.6	9506/739	Baseline serum magnesium level (mEq/L): 0.50–1.40; 1.50; 1.60; 1.70; 1.80; 1.90–2.60.	Serum magnesium was measured using the metallo-chromic dye calmagite, based on the procedure of Gindler and Heth.	Diabetes mellitus was defined as the presence of any of the following: 1) fasting glucose levels ≥ 7.0 mmol/L; 2) non-fasting glucose levels ≥11.1 mmol/L; 3) current use of diabetic medication; 4) a positive response to the question “Has a doctor ever told you that you had diabetes?” Individuals without diabetes at baseline who met any of these conditions at follow-up were considered to have incident cases of diabetes.	Age, gender, education, BMI, family history of diabetes, waist/hip ratio, sports index, alcohol consumption, diuretic use, serum calcium and potassium levels, and fasting insulin and glucose levels at baseline.	OR (95% CI):1.55 (1.01, 2.37);1.11 (0.76, 1.63);1.05 (0.73, 1.50);1.10 (0.78, 1.57);1.07 (0.74, 1.57);1.00 (Referent).*P*trend = 0.10.	9
Everett et al. (2006) [30], NHANES I, USA	25–74	NA	15.0	9784/690	Baseline serum magnesium level in quartiles (mEq/L): ≤1.60; 1.60–1.68; 1.69–1.77; ≥1.78.	Atomic absorption spectrophotometry	Incident diabetic cases were identified from nursing home and hospital records and death certificates. ICD-9 (250.0–250.9) representing diabetes was used to identify individuals who were admitted to a health care facility.	Age, gender, race, education, exercise, BMI, hypertension, and total cholesterol.	HR (95% CI):1.51 (1.12, 2.03);1.20 (0.88, 1.62);0.99 (0.73, 1.34);1.00 (Referent).	9
Guerrero-Romero et al. (2008) [31], The Mexican Diabetes Prevention study, Mexico	56.7 ± 11.9 (20–65)	NA	6.6	817/78	Baseline serum magnesium level in two groups (mmol/L): <0.74; ≥0.74.	Serum magnesium was measured by colorimetric method with the Data Pro Plus Random Access Clinical Analyzer (Arlington, TX, USA).	Diabetes mellitus was defined as the presence of any of the following: 1) 2-h post load serum glucose levels ≥11.1 mmol/L; 2) current use of diabetic medication(hypoglycemic drugs or insulin).	Age, gender, family history of diabetes, waist circumference, and HOMA-IR index.	RR (95% CI):2.54 (1.1, 4.1);1.00 (Referent).	7
Kieboom et al. (2017) [32], The Rotterdam Study, the Netherlands	64.7 ± 9.7	42.2	6.7	8555/806	Baseline serum magnesium level in two groups (mmol/L): ≤0.72; >0.72.	Serum magnesium was measured using a colorimetric endpoint method with the Roche/Hitachi Cobas c501 Analyzer (Roche Diagnostics, Indianapolis, IN, USA)	Incident diabetic cases were ascertained from general practioners’ records, hospital discharge letters and glucose measurements. Diabetes was defined as the presence of any of the following: 1) fasting glucose levels ≥ 7.0 mmol/L; 2) non-fasting glucose levels ≥11.1 mmol/L; 3) current use of blood glucose lowering medication.	Age, age^2^, gender, BMI, smoking status, alcohol use, total cholesterol: HDL-cholesterol ratio, history of hypertension, stroke and CHD, eGFR, serum levels of calcium and potassium, as well as use of diuretics	HR (95% CI):1.79 (1.16, 2.77);1.00 (Referent).Continuous(↓0.1 mmol/L):1.18 (1.04, 1.33).	9

*Abbreviations*: *ARIC* Atherosclerosis Risk in Communities, *BMI* body mass index, *CHD* coronary heart disease, *CI* confidence interval, *CVD* cardiovascular diseases, *DBP* diastolic blood pressure, *eGFR* estimated glomerular filtration rate, *HDL* high-density lipoprotein, *HOMA-IR* homeostasis model assessment for insulin resistance, *HR* hazards ratio, *IHD* ischemic heart disease, *MI* myocardial infarction, *NA* not available, *NHANES* National Health and Nutrition Examination Survey, *OR* odds ratio, *PREVEND* THE Prevention of Renal and Vascular End-Stage Disease Study, *RR* relative risk, *SBP* systolic blood pressure, *USA* the United States of America

*It was evaluated with the Newcastle-Ottawa scale

The average age at baseline was approximately 52 years old, and about 43.4% participants were male. All the included studies adjusted for age and sex, except for the studies that only included one gender. Also, the primary studies controlled for various potential confounders, including body mass index (*n* = 9) [11, 23, 25–30, 32] and/or other body composition variables such as waist/hip ratio (*n* = 3) [11, 24, 28] and waist circumference (*n* = 1) [31], smoking (*n* = 7) [23–27, 29, 32], alcohol consumption (*n* = 7) [11, 23–25, 27, 29, 32], physical activity (*n* = 6) [11, 23–25, 28, 30], and education(*n* = 5) [11, 24, 25, 28, 30]. Few studies adjusted for circulating levels of other nutrients such as calcium and potassium (*n* = 4) [11, 27, 29, 32] or estimated glomerular filtration rate (*n* = 2) [26, 32]. Ten out of the 11 included studies were assessed as high quality and only 1 yielded moderate quality (see Additional file 1: Table S2 in supplemental materials).

### Circulating magnesium levels and CHD risk

**Fig. 2:**
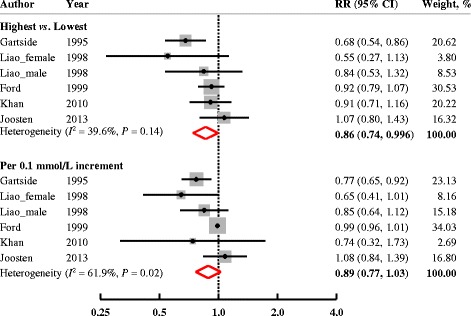
Multivariable-adjusted RRs (95% CIs) for incidence of CHD comparing highest to lowest, or per 0.1 mmol/L increment in circulating Mg levels from prospective cohort studies. The summary estimate was obtained by using a random-effects model. The dots indicate the adjusted RRs. The size of the shaded square is proportional to the weight of each study. The horizontal lines represent 95% CIs. The diamond markers indicate the pooled RRs. Abbreviations: CHD, coronary heart disease; CI, confidence interval; RR, relative risk

Five studies (6 cohorts) reported data for CHD (Fig. 2). The summary estimate was 0.86 (95% CI: 0.74, 0.996; *P* = 0.04). There was no significant heterogeneity among the studies (*I* ^2^ = 39.6%, *P* = 0.14) and no significant publication bias (Egger’s test: *P* = 0.48; Begg’s test: *P* = 0.57).

Dose-response analysis did not reveal a linear association between circulating Mg levels and incidence of CHD (RR, 0.89; 95% CI: 0.77, 1.03; *P*_trend_ = 0.10 with a 0.1 mmol/L increment in Mg levels). High heterogeneity was observed among the 6 included cohorts (*I* ^2^ = 61.9%, *P* = 0.02). There was no evidence indicating significant publication bias (Begg’s test, *P* = 0.14; Egger’s test, *P* = 0.57).

### Circulating magnesium levels and hypertension risk

**Fig. 3:**
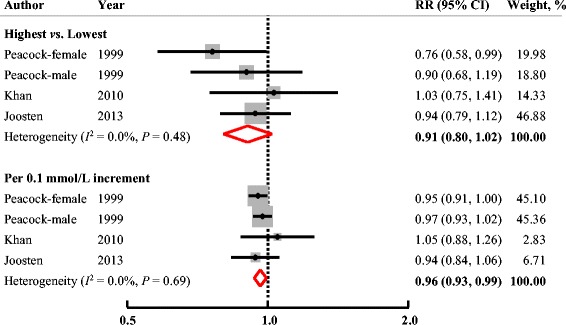
Multivariable-adjusted RRs (95% CIs) for incidence of hypertension comparing highest to lowest, or per 0.1 mmol/L increment in serum Mg levels from prospective cohort studies. The summary estimate was obtained by using a random-effects model. The dots indicate the adjusted RRs. The size of the shaded square is proportional to the weight of each study. The horizontal lines represent 95% CIs. The diamond markers indicate the pooled RRs. Abbreviations: CI, confidence interval; RR, relative risk

Three studies (4 cohorts) reported data on incidence of hypertension (Fig. 3). The pooled RR of incident hypertension was 0.91 (95% CI: 0.80, 1.02; *P* = 0.10) comparing the highest to the lowest circulating Mg levels. No significant heterogeneity among studies was found (*I* ^2^ = 0.0%, *P* = 0.48). Neither Egger’s (*P* = 0.86) nor Begg’s (*P* = 0.50) test indicated significant publication bias.

A significant inverse linear association was observed between circulating Mg levels and incidence of hypertension (RR, 0.96; 95% CI: 0.93, 0.99; *P*_trend_ = 0.02 with a 0.1 mmol/L increment in Mg levels). No significant heterogeneity across studies (*I* ^2^ = 0.0%, *P* = 0.69) and no evidence of publication bias were found (Egger’s test: *P* = 0.62; Begg’s test: *P* = 0.50).

### Circulating magnesium levels and diabetic risk

**Fig. 4:**
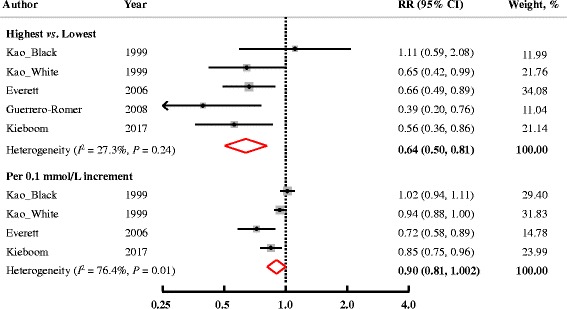
Multivariable-adjusted RRs (95% CIs) for incidence of T2DM comparing highest to lowest, or per 0.1 mmol/L increment in serum Mg levels from prospective cohort studies. The summary estimate was obtained by using a random-effects model. The dots indicate the adjusted RRs. The size of the shaded square is proportional to the weight of each study. The horizontal lines represent 95% CIs. The diamond markers indicate the pooled RRs. Abbreviations: CI, confidence interval; RR, relative risk; T2DM, type 2 diabetes mellitus

Four studies (5 cohorts) represented results on T2DM (Fig. 4). The pooled RR for incidence of T2DM comparing the highest to the lowest category of Mg levels was 0.64 (95% CI: 0.50, 0.81; *P* = 0.01). There was no significant heterogeneity among the studies (*I* ^2^ = 27.3%, *P* = 0.24), and no significant publication bias was found (Egger’s test: *P* = 0.94; Begg’s test: *P* = 0.46).

According to the available data from 3 cohorts, a non-significant linear association was observed (RR, 0.90; 95% CI: 0.81, 1.002; *P*_trend_ = 0.054 with a 0.1 mmol/L increment in circulating Mg levels). But, a high heterogeneity across studies was detected (*I* ^2^ = 76.4%, *P* = 0.01). No evidence of publication bias was found (Egger’s test: *P* = 0.26; Begg’s test: *P* = 0.31).

The funnel plots did not indicate publication bias for any pooling in this meta-analysis (See Additional file 1: Figure S1 in supplemental materials).

### Sensitivity analysis

When replacing a random-effects model with a fixed-effects model, the results were generally consistent, except that the linear association between circulating Mg levels and incidence of T2DM became statistically significant (0.94; 95% CI: 0.90, 0.98; *P* < 0.01) (see Additional file 1: Table S3 in supplemental materials).

Additional file 1: Table S4 (see supplemental materials) presents the influence of a single study on the overall associations of interest by omitting one study at each time from the pooled analysis. The categorical association between circulating Mg levels (highest vs. lowest) and incidence of CHD was attenuated to some extent by omitting Gartside et al. [23] (RR, 0.92; 95% CI: 0.82, 1.03; *P* = 0.16), Liao et al. [24] (female cohort: RR, 0.88; 95% CI: 0.76, 1.01; *P* = 0.07; male cohort: RR, 0.86; 95% CI: 0.73, 1.02; *P* = 0.08), Ford et al. [25] (RR, 0.83; 95% CI: 0.68, 1.02; *P* = 0.08), or Khan et al. [26] (RR, 0.84; 95% CI: 0.70, 1.02; *P* = 0.08). The linear association between circulating Mg levels and incidence of CHD was strengthened and became statistically significant (RR, 0.84; 95% CI: 0.71, 0.99; *P* = 0.04) when Ford et al. [25] was omitted.

No single study substantially changed the categorical association between circulating Mg levels (highest vs. lowest) and incidence of hypertension. However, when the female cohort from Peacock et al. [28] was omitted, the overall linear association between circulating Mg levels and incidence of hypertension was attenuated and became statistically non-significant (RR, 0.97; 95% CI: 0.93, 1.01; *P* = 0.20).

The overall categorical association between circulating Mg levels (highest vs. lowest) and incidence of T2DM persisted with excluding any single study each time in the meta-analysis, while the linear association between circulating Mg levels and incidence of T2DM was strengthened and became statistically significant when Kao et al. [11] (RR, 0.86; 95% CI: 0.75, 0.97; *P* = 0.02) was omitted in the meta-analysis.

In addition, when we transformed OR to RR in one study for hypertension [28], and the other one for diabetes [11], the related results were not materially changed (data not shown).

## Discussion

Findings from this meta-analysis of prospective cohort studies suggested that circulating Mg levels were inversely associated with incidence of CHD, hypertension and T2DM. The present study is complementary to the previous systematic reviews on dietary Mg intake and provides additional evidence in support of the potential beneficial effect of Mg on CVD and diabetes.

### Comparison with previous reviews

Although no previous meta-analyses specifically focused on CHD, several meta-analyses have studied the association of circulating Mg levels and CVD risk [13, 34]. One meta-analysis of prospective studies published in 2013, which combined CVD incidence and mortality, reported that every 0.2 mmol/L increment in circulating Mg levels was associated with a 30% lower risk of CVD and a 17% lower risk of ischemic heart disease [13]. Another meta-analysis also published in 2013 found that the pooled RR of total CVD events (including CVD incidence and mortality) was 23% lower comparing the highest to the lowest serum Mg levels [34]. Also, a recent meta-analysis observed a significant but heterogeneous inverse association between serum Mg and metabolic syndrome [40]. Another recent meta-analysis investigated dietary Mg intake, but not circulating Mg level, in relation to type 2 diabetes and other CVD endpoints [41]. By contrast, we focused on research about CHD events and our findings contribute additional information to the literature.

To the best of our knowledge, this is the first systematic review to summarize studies on circulating Mg levels and incidence of hypertension. Our findings are generally consistent with previous reviews and meta-analyses of interventional studies on Mg supplementation [42–44]. For example, one meta-analysis of clinical trials found that Mg supplementation decreased systolic blood pressure (SBP) (3–4 mmHg) and diastolic blood pressure (DBP) (2–3 mmHg) in both normotensive and hypertensive individuals. [43] Another meta-analysis found that Mg supplementation significantly reduced SBP (18.7 mmHg) and DBP (10.9 mmHg) in hypertensive patients with SBP > 155 mmHg [44].

Several meta-analyses examined the association of dietary Mg intake and diabetes risk [9, 45–47]. For example, an updated meta-analysis of prospective cohort studies published in 2015 found that Mg intake was significantly and inversely associated with risk of T2DM in a nonlinear dose-response manner (*P*_nonlinearity_ = 0.003) [45]. In addition, a most-recent published systematic review and meta-analysis of randomized clinical trials reported that oral Mg supplementation improved insulin-sensitivity parameters in those who were at high risk for diabetes [48]. Similarly, findings from our meta-analysis of circulating Mg levels and risk of T2DM are supplementary to previous systematic reviews on dietary Mg intake or Mg supplementation.

### Strengths and limitations

Our meta-analysis has several strengths. First, this is the first up-to-date meta-analysis examining the associations between circulating Mg levels and incidence of CHD, hypertension, and T2DM together in the general population. Second, this meta-analysis was based on prospective cohort studies of high or moderate quality from various populations, which reduced the likelihood that our findings were substantially biased by the inherent limitations in the primary studies. Third, the combined sample size was relatively large and the long duration of follow-up enabled us to examine the long-term association. Considering that randomized controlled trials are relatively small and include short follow-up periods, our data provide important supplementary information to the literature. Finally, our conclusions are strengthened by generally consistent findings from both categorical and linear analyses, as well as the robust findings from the sensitivity analyses.

Some limitations also need to be acknowledged. Although we found significant associations, the present meta-analysis was based on 10 published cohort studies. The limited data sources not only restricted us from doing stratified or subgroup analyses, but also may potentially lead to over- or under-estimation of the true associations. Second, similar to other meta-analyses of observational studies, the inherent limitations in primary studies may bias the pooled results, though various potential confounders were considered in the original studies. Of note, factors such as family history of chronic diseases and medications related to Mg metabolism were seldom mentioned in the included studies. Third, the possibility of misclassification from both exposure and outcome could not be completely excluded. However, objective biomarkers were used for Mg assessment, and objective evidence (e.g., medical records and death certificates) was used for outcome assessment in most of the included studies. Fourth, although we did not observe evidence of publication bias, the likelihood could not be completely excluded due to publications in other languages. Finally, we realize that circulating Mg may not be the best biomarker, thus alternative biomarkers (e.g., red blood cell Mg and ionized Mg) are definitely needed in future studies depending on the scale of the study and other considerations such as budget and ethics.

### Potential mechanisms

Mg is a co-factor of more than 350 essential metabolic reactions. Most importantly, as a component of the Mg-adenosine triphosphate complex, Mg is involved with all phosphate transfer reactions [6, 7, 49]. Experimental studies show that Mg could: 1) regulate vascular smooth muscle tone through modulating calcium entry and intracellular signal pathways [50, 51]; and 2) regulate endothelial function through adjusting the synthesis and release of vasodilatory prostacyclin and nitric oxide [52, 53]. These vascular effects of Mg form the link between its deficiency and the pathogenesis of CHD and hypertension. Studies also found that Mg inhibited experimental arterial thrombus formation by inhibition of platelet aggregation [54], and Mg deficiency resulted in inflammation in various parts of the heart [55] and acceleration of the atherosclerotic process [56], which were related to the development of CHD. In recent meta-analyses and reviews, researchers found that low diet Mg intake and hypomagnesemia were correlated with low-grade inflammation and oxidative stress [57–59], both of which are known to be part of the pathogenesis of chronic diseases such as CHD and T2DM.

In addition to Mg involvement in glucose metabolism, including the glycolytic pathway and the Krebs cycle, studies have shown that Mg is essential in insulin signal transduction through the activation of the *β*-subunit of the tyrosine kinase domain of the insulin receptor, which is a critical step in the trans-membrane signaling cascade of the insulin reaction [6, 7, 60]. Animal experiments found that Mg deficiency was associated with reduced glucose uptake and utilization in insulin-sensitive tissues, thus promoting insulin resistance and the development of diabetes [25, 61]. Furthermore, randomized controlled trials including non-diabetic individuals found improvement in insulin sensitivity together with increased levels of serum Mg after Mg supplementation [62–64].

## Conclusions

In conclusion, this meta-analysis of prospective cohort studies found that circulating Mg was inversely associated with incidence of CHD, hypertension, and T2DM. Findings of this meta-analysis are supplementary to previous reviews on dietary Mg intake and risk of CVD and diabetes. Further studies are needed to provide more solid evidence, and to elucidate the dose-response relationship and to explore the optimal range of circulating Mg concentrations in terms of prevention of CHD, hypertension, and T2DM.

## Additional file

Additional file 1: Table S1. PRISMA checklist. **Table S2.** Quality assessment using Newcastle-Ottawa quality assessment scale for the studies included in the meta-analysis. **Table S3.** Multivariable adjusted association of circulating magnesium levels with incidence of CHD, hypertension, and T2DM using a fixed-effects model: a sensitivity analysis. **Table S4.** Influence of a single study on the pooled association of circulating magnesium levels with incidence of CHD, hypertension, and T2DM using a random-effects model: a sensitivity analysis. **Figure S1.** Funnel plots with pseudo 95% CLs for six pooling in this meta-analysis. (DOC 202 kb)

## Abbreviations

CHD: Coronary heart diseases
CI: Confidence interval
CVD: Cardiovascular diseases
DBP: Diastolic blood pressure
HR: Hazard ratio
Mg: Magnesium
NOS: The Newcastle-Ottawa quality assessment scale
OR: Odds ratio
PRISMA: The preferred reporting items for systematic reviews and meta-analyses
RR: Relative risk
SBP: Systolic blood pressure
T2DM: Type 2 diabetes
