# Supplementary material for: Circulating magnesium levels and incidence of coronary heart diseases, hypertension, and type 2 diabetes mellitus: a meta-analysis of prospective cohort studies
Source: Nutr J. 2017 Sep 19;16:60. doi: 10.1186/s12937-017-0280-3 (PMC5606028; doi:10.1186/s12937-017-0280-3)
Supplement: Additional file 1: Table S1. — PRISMA checklist. Table S2. Quality assessment using Newcastle-Ottawa quality assessment scale for the studies included in the meta-analysis. Table S3. Multivariable adjusted association of circulating magnesium levels with incidence of CHD, hypertension, and T2DM using a fixed-effects model: a sensitivity analysis. Table S4. Influence of a single study on the pooled association of circulating magnesium levels with incidence of CHD, hypertension, and T2DM using a random-effects model: a sensitivity analysis. Figure S1. Funnel plots with pseudo 95% CLs for six pooling in this meta-analysis. (DOC 202 kb) [file 12937_2017_280_MOESM1_ESM.doc]

Table S1

PRISMA checklist

| **Section/topic** | **#** | **Checklist item** | **Reported on page #** |
| --- | --- | --- | --- |
| **TITLE** | | |  |
| Title | 1 | Identify the report as a systematic review, meta-analysis, or both. | 1 |
| **ABSTRACT** | | |  |
| Structured summary | 2 | Provide a structured summary including, as applicable: background; objectives; data sources; study eligibility criteria, participants, and interventions; study appraisal and synthesis methods; results; limitations; conclusions and implications of key findings; systematic review registration number. | 2-3 |
| **INTRODUCTION** | | |  |
| Rationale | 3 | Describe the rationale for the review in the context of what is already known. | 4-5 |
| Objectives | 4 | Provide an explicit statement of questions being addressed with reference to participants, interventions, comparisons, outcomes, and study design (PICOS). | 4-5 |
| **METHODS** | | |  |
| Protocol and registration | 5 | Indicate if a review protocol exists, if and where it can be accessed (e.g., Web address), and, if available, provide registration information including registration number. | 6 |
| Eligibility criteria | 6 | Specify study characteristics (e.g., PICOS, length of follow-up) and report characteristics (e.g., years considered, language, publication status) used as criteria for eligibility, giving rationale. | 6-7 |
| Information sources | 7 | Describe all information sources (e.g., databases with dates of coverage, contact with study authors to identify additional studies) in the search and date last searched. | 6 |
| Search | 8 | Present full electronic search strategy for at least one database, including any limits used, such that it could be repeated. | 6 |
| Study selection | 9 | State the process for selecting studies (i.e., screening, eligibility, included in systematic review, and, if applicable, included in the meta-analysis). | 6-7 |
| Data collection process | 10 | Describe method of data extraction from reports (e.g., piloted forms, independently, in duplicate) and any processes for obtaining and confirming data from investigators. | 6-7 |
| Data items | 11 | List and define all variables for which data were sought (e.g., PICOS, funding sources) and any assumptions and simplifications made. | 7-8 |
| Risk of bias in individual studies | 12 | Describe methods used for assessing risk of bias of individual studies (including specification of whether this was done at the study or outcome level), and how this information is to be used in any data synthesis. | 8-9 |
| Summary measures | 13 | State the principal summary measures (e.g., risk ratio, difference in means). | 8 |
| Synthesis of results | 14 | Describe the methods of handling data and combining results of studies, if done, including measures of consistency (e.g., I^2^) for each meta-analysis. | 8-9 |
| Risk of bias across studies | 15 | Specify any assessment of risk of bias that may affect the cumulative evidence (e.g., publication bias, selective reporting within studies). | 8-9 |
| Additional analyses | 16 | Describe methods of additional analyses (e.g., sensitivity or subgroup analyses, meta-regression), if done, indicating which were pre-specified. | 8-9 |
| **RESULTS** | | |  |
| Study selection | 17 | Give numbers of studies screened, assessed for eligibility, and included in the review, with reasons for exclusions at each stage, ideally with a flow diagram. | 9  Fig. 1 |
| Study characteristics | 18 | For each study, present characteristics for which data were extracted (e.g., study size, PICOS, follow-up period) and provide the citations. | 9-10,  Table 1 |
| Risk of bias within studies | 19 | Present data on risk of bias of each study and, if available, any outcome level assessment (see item 12). | 10-12 |
| Results of individual studies | 20 | For all outcomes considered (benefits or harms), present, for each study: (a) simple summary data for each intervention group (b) effect estimates and confidence intervals, ideally with a forest plot. | 10-12,  Fig. 2-4 |
| Synthesis of results | 21 | Present results of each meta-analysis done, including confidence intervals and measures of consistency. | 10-12  Fig. 2-4 |
| Risk of bias across studies | 22 | Present results of any assessment of risk of bias across studies (see Item 15). | 10-12 |
| Additional analysis | 23 | Give results of additional analyses, if done (e.g., sensitivity or subgroup analyses, meta-regression [see Item 16]). | 10-12  Table S3,S4 |
| **DISCUSSION** | | |  |
| Summary of evidence | 24 | Summarize the main findings including the strength of evidence for each main outcome; consider their relevance to key groups (e.g., healthcare providers, users, and policy makers). | 13 |
| Limitations | 25 | Discuss limitations at study and outcome level (e.g., risk of bias), and at review-level (e.g., incomplete retrieval of identified research, reporting bias). | 15-16 |
| Conclusions | 26 | Provide a general interpretation of the results in the context of other evidence, and implications for future research. | 17 |
| **FUNDING** | | |  |
| Funding | 27 | Describe sources of funding for the systematic review and other support (e.g., supply of data); role of funders for the systematic review. | 18 |

*From:*  Moher D, Liberati A, Tetzlaff J, Altman DG, The PRISMA Group (2009). Preferred Reporting Items for Systematic Reviews and Meta-Analyses: The PRISMA Statement. PLoS Med 6(7): e1000097. doi:10.1371/journal.pmed1000097

Table S2

Quality assessment using Newcastle-Ottawa quality assessment scale for the studies included in the meta-analysis

| **Source** | **Selection^*^** | **Comparability^†^** | **Outcome^‡^** | **Quality** |
| --- | --- | --- | --- | --- |
| Gartside et al. (1995) , The NHANES Study, USA | ★★★★ | ★★ | ★★★ | High |
| Liao et al., (1998) , The ARIC Study, USA | ★★★★ | ★★ | ★★★ | High |
| Ford et al., (1999) , The NHANES Study, USA | ★★★★ | ★★ | ★★★ | High |
| Kao et al., (1999) , The ARIC Study, USA | ★★★★ | ★★ | ★★★ | High |
| Peacock et al., (1999) , The ARIC Study, USA | ★★★★ | ★★ | ★★★ | High |
| Everett et al., (2006) , NHANES I, USA | ★★★★ | ★★ | ★★★ | High |
| Guerrero-Romero et al., (2008 ), The Mexican Diabetes Prevention study, Mexico | ★★★ | ★★ | ★★ | Moderate |
| Khan et al., (2010) , The Framingham offspring Study, USA | ★★★★ | ★★ | ★★★ | High |
| Joosten et al., (2013 a) , The PREVEND study, The Netherlands | ★★★★ | ★★ | ★★★ | High |
| Joosten et al., (2013 b) , The PREVEND study, The Netherlands | ★★★★ | ★★ | ★★★ | High |
| Kieboom BC et al.,(2017), The Rotterdam Study, the Netherlands | ★★★★ | ★★ | ★★★ | High |

Abbreviations: ARIC: Atherosclerosis Risk in Communities; NHANES: National Health and Nutrition Examination Survey; PREVEND: THE Prevention of Renal and Vascular End-Stage Disease Study; USA: the United States of America.

^*^Stars awarded for representativeness of the cohort, selection of the cohort, the assessment of exposure, and the demonstration that the outcome of interest was not present at start of study. A maximum of 4 stars is to be awarded.

^†^Stars awarded for adjustment of potential confounders. A maximum of 2 stars is to be awarded.

^‡^Stars awarded for assessment of outcome, length of follow-up, and adequacy of follow up of cohort. A maximum of 3 stars is to be awarded.

Table S3

Multivariable adjusted association of circulating magnesium levels with incidence of CHD, hypertension, and T2DM using a fixed-effects model: a sensitivity analysis

| **Outcome** | **RR (95%CI)** | |
| --- | --- | --- |
|  | **Highest *vs*. Lowest** | **Per 0.1 mmol/L increment** |
| CHD | 0.87 (0.79, 0.97) | 0.98 (0.96, 1.01) |
| Hypertension | 0.91 (0.80, 1.02) | 0.96 (0.93, 0.99) |
| T2DM | 0.64 (0.50, 0.78) | 0.94 (0.90, 0.98) |

Abbreviations: CHD, coronary heart disease; CI, confidence interval; RR, relative risk; T2DM, type 2 diabetes mellitus.

Table S4

Influence of a single study on the pooled association of circulating magnesium levels with incidence of CHD, hypertension, and T2DM using a random-effects model: a sensitivity analysis

| **Outcome** | **Study/cohort omitted** | **RR (95% CI)** | |
| --- | --- | --- | --- |
|  |  | **Highest *vs*. Lowest** | **Per 0.1 mmol/L increment** |
| CHD | **None** | **0.86 (0.74, 0.996)** | **0.89 (0.77, 1.03)** |
|  | Gartside et al.(1995) | 0.92 (0.82, 1.03) | 0.95 (0.84, 1.07) |
|  | Liao et al._female, (1998) | 0.88 (0.76, 1.01) | 0.91 (0.80, 1.05) |
|  | Liao et al._male, (1998) | 0.86 (0.73, 1.02) | 0.89 (0.75, 1.05) |
|  | Ford et al. (1999) | 0.83 (0.68, 1.02) | **0.84 (0.71, 0.99)** |
|  | Khan et al.(2010) | 0.84 (0.70, 1.02) | 0.89 (0.76, 1.04) |
|  | Joosten et al. (2013 a) | 0.83 (0.71, 0.96) | 0.84 (0.71, 1.01) |
| Hypertension | **None** | **0.91 (0.80, 1.02)** | **0.96 (0.93, 0.99)** |
|  | Peacock et al._female, (1999) | 0.95 (0.83, 1.08) | 0.97 (0.93, 1.01) |
|  | Peacock et al._male, (1999) | 0.90 (0.78, 1.05) | 0.96 (0.92, 0.996) |
|  | Khan et al.(2010) | 0.89 (0.79, 1.01) | 0.96 (0.93, 0.99) |
|  | Joosten et al.(2013 b) | 0.88 (0.74, 1.04) | 0.97 (0.94, 0.996) |
| T2DM | **None** | **0.64 (0.50, 0.81)** | **0.90 (0.81, 1.002)** |
|  | Kao et al._black, (1999) | 0.60 (0.49, 0.74) | 0.86 (0.75, 0.97) |
|  | Kao et al._white, (1999) | 0.64 (0.46, 0.88) | 0.87 (0.72, 1.04) |
|  | Everett et al.(2006) | 0.63 (0.44, 0.89) | 0.94 (0.86, 1.03) |
|  | Guerrero-Romero et al. (2008) | 0.67 (0.54, 0.83) | -- |
|  | Kieboom BC et al.,(2017) | 0.66 (0.49, 0.90) | 0.92 (0.81, 1.04) |

Abbreviations: CHD, coronary heart disease; CI, confidence interval; RR, relative risk; T2DM, type 2 diabetes mellitus

Fig. S1

Funnel plots with pseudo 95% CLs for six pooling in this meta-analysis. The dots indicate the adjusted RRs. The vertical line is plotted at the value of the fixed effect summary estimate in log scale [i.e., ln(RR)]. The dash lines represent 95% CLs. Abbreviations: CHD, coronary heart disease; CL, confidence limit; RR, relative risk; T2DM, type 2 diabetes mellitus.

**References**

1. Gartside PS, Glueck CJ. The important role of modifiable dietary and behavioral characteristics in the causation and prevention of coronary heart disease hospitalization and mortality: the prospective NHANES I follow-up study**.** J Am Coll Nutr. 1995; 14**:**71-9.

2. Liao F, Folsom AR, Brancati FL. Is low magnesium concentration a risk factor for coronary heart disease? The Atherosclerosis Risk in Communities (ARIC) Study**.** Am Heart J. 1998; 136**:**480-90.

3. Ford ES. Serum magnesium and ischaemic heart disease: findings from a national sample of US adults**.** Int J Epidemiol. 1999; 28**:**645-51.

4. Kao WH, Folsom AR, Nieto FJ, Mo JP, Watson RL, Brancati FL. Serum and dietary magnesium and the risk for type 2 diabetes mellitus: the Atherosclerosis Risk in Communities Study**.** Arch Intern Med. 1999; 159**:**2151-9.

5. Peacock JM, Folsom AR, Arnett DK, Eckfeldt JH, Szklo M. Relationship of serum and dietary magnesium to incident hypertension: the Atherosclerosis Risk in Communities (ARIC) Study**.** Ann Epidemiol. 1999; 9**:**159-65.

6. Everett CJ, King DE. Serum magnesium and the development of diabetes**.** Nutrition. 2006; 22**:**679.

7. Guerrero-Romero F, Rascon-Pacheco RA, Rodriguez-Moran M, de la Pena JE, Wacher N. Hypomagnesaemia and risk for metabolic glucose disorders: a 10-year follow-up study**.** Eur J Clin Invest. 2008; 38**:**389-96.

8. Khan AM, Sullivan L, McCabe E, Levy D, Vasan RS, Wang TJ. Lack of association between serum magnesium and the risks of hypertension and cardiovascular disease**.** Am Heart J. 2010; 160**:**715-20.

9. Joosten MM, Gansevoort RT, Mukamal KJ, van der Harst P, Geleijnse JM, Feskens EJ, Navis G, Bakker SJ, Group PS. Urinary and plasma magnesium and risk of ischemic heart disease**.** Am J Clin Nutr. 2013; 97**:**1299-306.

10. Joosten MM, Gansevoort RT, Mukamal KJ, Kootstra-Ros JE, Feskens EJ, Geleijnse JM, Navis G, Bakker SJ, Group PS. Urinary magnesium excretion and risk of hypertension: the prevention of renal and vascular end-stage disease study**.** Hypertension. 2013; 61**:**1161-7.

11. Kieboom BC, Ligthart S, Dehghan A, Kurstjens S, de Baaij JH, Franco OH, Hofman A, Zietse R, Stricker BH, Hoorn EJ. Serum magnesium and the risk of prediabetes: a population-based cohort study**.** Diabetologia. 2017; 60**:**843-53.
